# COVID‐19 associated EBV reactivation and effects of ganciclovir treatment

**DOI:** 10.1002/iid3.597

**Published:** 2022-03-10

**Authors:** Mei Meng, Sheng Zhang, Xuan Dong, Wenqing Sun, Yunfeng Deng, Wenzhe Li, Ranran Li, Djillali Annane, Zhixiong Wu, Dechang Chen

**Affiliations:** ^1^ Department of Critical Care Medicine, Ruijin Hospital Shanghai Jiao Tong University School of Medicine Shanghai China; ^2^ Tuberculosis and Respiratory Department Wuhan Infectious Disease Hospital Wuhan China; ^3^ Department of Intensive Care Unit Shandong Provincial Chest Hospital Jinan China; ^4^ Katharine Hsu International Research Center of Human Infectious Diseases Shandong Provincial Chest Hospital Jinan China; ^5^ General Intensive Care Unit, Laboratory of Inflammation and Infection U1173, Raymond Poincaré Hospital (APHP) University of Versailles SQY/INSERM Garches France; ^6^ Department of Surgical Intensive Care Unit Huadong Hospital Affiliated to Fudan University Shanghai China

**Keywords:** COVID‐19, EBV reactivation, ganciclovir, mortality

## Abstract

**Background:**

Systemic reactivation of Epstein–Barr virus (EBV) may occur in novel coronavirus disease 2019 (COVID‐19) caused by the severe acute respiratory syndrome coronavirus‐2 (SARS‐CoV‐2). However, the clinical consequences of EBV reactivation remain uncertain.

**Methods:**

In this retrospective study, we screened 1314 patients with confirmed COVID‐19 who died or were discharged between January 1, 2020 and March 12, 2020, in Wuhan Infectious Disease Hospital, Wuhan, China. Patients who had complete data for EBV serology and cytomegalovirus (CMV) serology were eligible. Serum levels of viral capsid antigen (VCA)‐immunoglobulin G (IgG), Epstein–Barr nuclear antigen‐IgG, VCA‐IgM, early antigen (EA)‐IgG, CMV‐IgG, and CMV‐IgM were compared between survivors and nonsurvivors. Dynamic changes of laboratory tests and outcomes were compared in patients with and without ganciclovir treatment. We used 1:1 matching based on age, gender, and illness severity to balance baseline characteristics.

**Results:**

EBV reactivation was present in 55 of 217 patients. EBV reactivation was associated with age (57.91 [13.19] vs. 50.28 [12.66] years, *p* < .001), female gender (31 [56%] vs. 60 [37%], *p* = .02). Patients with EBV reactivation have statistically nonsignificant higher mortality rate (12 [22%] vs. 18 [11%], *p* = .08). EA‐IgG levels were significantly higher in nonsurvivors than in survivors (median difference: −0.00005, 95% confidence interval, CI [−3.10, 0.00], *p* = .05). As compared to patients with COVID‐19 who did not receive ganciclovir therapy, ganciclovir‐treated patients had improved survival rate (0.98, 95% CI [0.95, 1.00] vs. 0.88, 95% CI [0.81, 0.95], *p* = .01). Hemoglobin (*p* < .001) and prealbumin (*p* = .02) levels were significantly higher in ganciclovir‐treated patients.

**Conclusion:**

A high proportion of COVID‐19 patients had EBV reactivation that may be associated with an increased risk of death. Whether treatment with ganciclovir may decrease the mortality of COVID‐19 patients complicated with EBV reactivation warrants to be addressed in a placebo‐controlled randomized trial in the future.

## Graphical abstract

A high proportion of coronavirus disease 2019 (COVID‐19) patients had Epstein–Barr virus (EBV) reactivation that may be associated with an increased risk of death. Whether treatment with ganciclovir may decrease the mortality of COVID‐19 patients complicated with EBV reactivation warrants to be addressed in a placebo‐controlled randomized trial in the future.

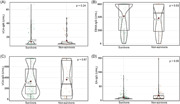

## 1 INTRODUCTION

Severe acute respiratory syndrome coronavirus‐2 (SARS‐CoV‐2) related pandemic places an unprecedented burden on about 185 countries. Owing to the lack of vaccine and of effective antiviral therapy, and to SARS‐CoV‐2 virus contagiousness, the spread of the novel coronavirus disease 2019 (COVID‐19) remains uncontrolled.^1^ Therefore, increasing knowledge of the mechanisms of COVID‐19, identifying relevant therapeutic targets, and screening the benefit and risks of available drugs are top priorities.

About 16% of patients with COVID‐19 will develop critical illness,^2^ and crude mortality rates in these patients ranged from 11% to 60%.^3^, ^4^, ^5^ Typically, patients become severely hypoxic within 7–10 days from the onset of the first symptoms, followed by multiple organ dysfunctions, including cardiac, kidney and liver injury, gastrointestinal bleeding, and disseminated intravascular coagulopathy (DIC).^6^, ^7^ Lymphocytopenia occurs at an early stage of COVID‐19 and correlates to the severity of illness.^2^, ^8^ Acquired impaired immune function may contribute to the pathogenesis of COVID‐19. Epstein–Barr virus (EBV) is a common herpesvirus that causes latent infection in 90% adults.^9^ The level of herpesvirus replication correlates to host immune status. During sepsis, latent reactivation of EBV may occur in 50% of cases and may contribute to poor clinical outcome.^10^ Reactivation of EBV may be witnessed by serum concentrations of viral capsid antigen‐immunoglobulin G (VCA‐IgG) and early antigen IgG (EA‐IgG).^11^ SARS‐CoV‐2‐associated sepsis may increase the risk of EBV reactivation suggesting potential advantages of antiherpes therapy. As a chemically synthesized guanine analogue, ganciclovir is commonly used to treat acute infection and reactivation of EBV in patients with immune deficiency.^12^

This study aimed at investigating the incidence and consequences of EBV reactivation and the effects on survival of ganciclovir in patients with COVID‐19.

## 2 METHODS

### 2.1 Study design and participants

This was a retrospective study carried out at a single tertiary care center (Infectious Disease Hospital, Wuhan, China) and approved by the Research Ethics Committee of Wuhan Infectious Disease Hospital (KY‐2020‐03‐01). From January 1, 2020 to March 12, 2020, 1314 adults with confirmed COVID‐19 according to the clinical guidelines of China and WHO were included.

### 2.2 Data collection

Data collection used a specific and predefined case report form and was conducted by the critical care clinical trials group of Wuhan Infectious Disease Hospital and Shanghai Jiaotong University, School of Medicine, Ruijin Hospital. Data recordings were checked by a second party and any discrepancy was solved by a third researcher.

We recorded demographic data and past medical history. We also recorded on admission and daily during hospitalization clinical symptoms, physical examination, vital signs, acute physiology and chronic health evaluation score II (APACHEII) scores and sequential organ failure assessment (SOFA) scores, laboratory tests, imaging (computed tomography, chest X‐ray). We recorded ganciclovir therapy according to serological evidence in some patients suggesting that high risk of EBV reactivated. While pharmaceutical interventions (antiviral, antibiotic, glucocorticoid, and blood products), oxygen therapy, and organ support therapy (renal replacement therapy and extracorporeal membrane oxygenation) were documented. In addition, APACHEII and SOFA scores were obtained in all patients at least once every five days during hospitalization.

Laboratory tests included leukocytes, lymphocytes, and platelets counts, levels of hemoglobin, interleukin‐6 (IL‐6), C‐reactive protein (CRP), serum creatinine, ferritin, triglyceride, fibrinogen, erythrocyte sedimentation rate (ESR), EBV serology, and cytomegalovirus (CMV) serology were performed according to physicians' instruction. During the outbreak of COVID‐19, a large number of patients needed polymerase chain reaction (PCR) testing to detect SARS‐CoV‐2 nucleic acid, which resulted in a serious shortage of humans and resources for doing PCR testing. Therefore, only a few patients have been PCR tested for EBV and CMV DNA replication. We chose serological results as evidence to observe whether EBV or CMV reactivation according to existing research literature. EBV reactivation was defined as VCA‐IgG was above the normal level and/or EA‐IgG tested positive.^11^ VCA‐IgG, EBV nuclear antigen IgG (EBNA‐IgG), VCA‐IgM, EA‐IgG, CMV‐IgG, and CMV‐IgM were measured by commercial ELISA assay (Diasorin Liaison®) according to manufacturer's instructions as previous research.^13^

### 2.3 Definitions

Criteria for COVID‐19 included symptoms and positive reverse transcriptase (RT)‐PCR assays were in accordance with Chinese guidelines (sixth version). The illness severity was classified as mild, moderate, severe, critical according to the Chinese guidelines for COVID‐19.^7^ Acute respiratory distress syndrome was defined according to the Berlin definition.^14^ Acute kidney injury was defined according to the Kidney Disease: Improving Global Outcomes classification.^15^ Platelet counts, prothrombin time test, and the levels of fibrin/fibrinogen degradation products were used for the diagnosis of DIC.^16^ Sepsis and septic shock were defined according to the Third International Consensus Definition for Sepsis and Septic Shock.^17^ EB reactivation was defined as positive EA‐IgG (≥10 U/ml) and VCA‐IgG (≥20 U/ml) simultaneously. A positive VCA‐IgG (≥20 U/ml) was considered as evidence for past infection and indicated immunity, and a positive VCA‐IgM (≥20 U/ml) finding was indicative of current EBV infection. EBNA‐IgG (≥5 U/ml) revealed past infection with EBV.^18^ CMV‐IgM (≥18 U/ml) with or without CMV‐IgG (≥12 U/ml) was considered as evidence for an active primary CMV infection or a reactivated latent infection.^19^

### 2.4 Outcomes

The primary outcome was 28‐day mortality. The secondary outcome included the prevalence of positive EBV serology, CMV serology between nonsurvivors and survivors. Safety outcomes included kidney dysfunction assessed by serum creatinine and bone marrow suppression based on leukocyte count.

### 2.5 Statistical analysis

We first compared the prevalence of positive EBV serology (VCA‐IgG, EBNA‐IgG, VCA‐IgM, and EA‐IgG), CMV serology (CMV‐IgG and CMV‐IgM) between survivors and nonsurvivors. Then, we compared the demographic characteristics and laboratory parameters in COVID‐19 patients with and without EBV reactivation. Then, we compared patients treated with versus without ganciclovir. Ganciclovir‐treated patients were matched to controls, 1:1, based on age, gender, the severity of illness (APACHEII and SOFA scores).

Continuous data were expressed as mean and standard deviation, or median and interquartile range. Categorical variables were expressed as numbers and percentages. Comparison of continuous data used student *t*‐test or Wilcoxon rank‐sum test. Comparison of categorical data used *χ* ^2^ test or Fisher exact test, as appropriate. Differences in distributions of continuous data by grouping variables (survivors vs. nonsurvivors) were reported using differences with 95% confidence intervals (CIs). Dynamic changes of laboratory parameters between the ganciclovir treatment group and the nonganciclovir treatment group were compared by a two‐way analysis of variance with repeated measures. Kaplan–Meier plot and log‐rank test were used for survival analysis. A two‐sided *p *< .05 was considered statistically significant. Statistical analyses were performed using the R software (version 3.6.2).

## 3 RESULTS

Among the 1314 COVID‐19 patients admitted to Wuhan Infectious Disease Hospital by March 12th, 2020, 1097 patients without data on EBV and CMV serological results were excluded, retaining 217 patients for subsequent analysis, of whom 30 (13.82%) died and 187 (86.18%) were discharged alive. Among the 217 patients, 55 (25.3%) had EBV reactivation. When compared to patients without EBV reactivation, patients with EBV reactivation were older (57.91 [13.19] vs. 50.28 [12.66] years, *p* < .001), and there were more women (31 [56%] vs. 60 [37%], *p* = .02), and numerically more deaths (12 [22%] vs. 18 [11%], *p* = .08).

### 3.1 EBV serology and CMV serology in patients with COVID‐19

**FIGURE 1:**
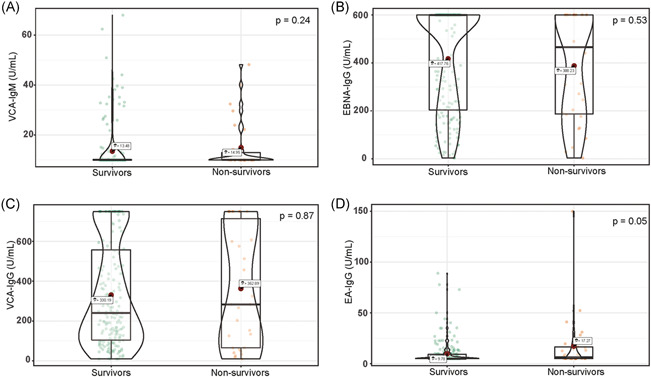
Violin plot of VCA‐IgM, EBNA‐IgG, VCA‐IgG, and EA‐IgG concentration among survivors and nonsurvivors. EA‐IgG, EBV early antigens‐IgG; EBNA‐IgG, EBV nuclear antigens‐IgG; EBV, Epstein–Barr virus; IgG, immunoglobulin G; VCA‐IgG, EBV viral capsid antigen‐IgG; VCA‐IgM, EBV viral capsid antigen‐IgM

**FIGURE 2:**
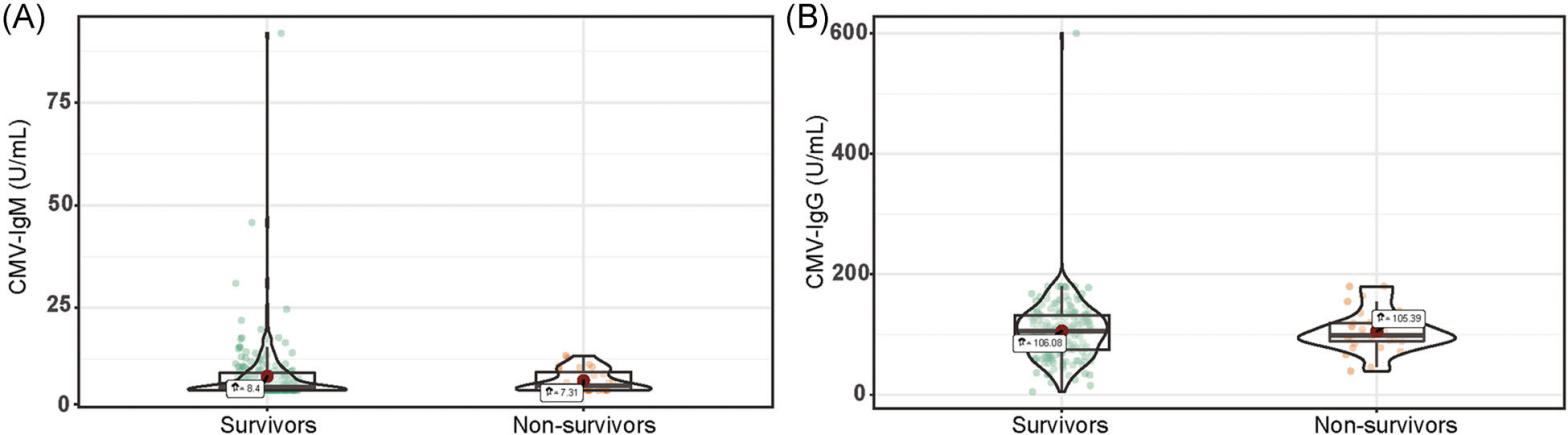
Violin plot of CMV‐IgM and CMV‐IgG concentration among survivors and nonsurvivors. CMV‐IgG, cytomegalovirus immunoglobulin G; CMV‐IgM, cytomegalovirus immunoglobulin M

There were no significant differences between survivors and nonsurvivors for the levels of VCA‐IgM (median difference: −0.00004, 95% CI [−0.00006, 0.00006] U/ml, *p* = .24), EBNA‐IgG (median difference: −0.00004, 95% CI [−0.00005, 37.00] U/ml, *p* = .53), VCA‐IgG (median difference: −0.00001, 95% CI [−106.00, 65.70] U/ml, *p* = .87) (Figure 1). Whereas EA‐IgG levels were higher in nonsurvivors than in survivors (median difference: −0.00005, 95% CI [−3.10, 0.00] U/ml, *p* = .05). There was no significant difference between survivors and nonsurvivors in serum concentrations of CMV‐IgM (median difference: −0.00002, 95% CI [−0.54, 0.40] U/ml, *p* = .88) and CMV‐IgG (median difference: 0.00004, 95% CI [−15.80, 15.40]  U/ml, *p* = .98) (Figure 2).

### 3.2 Comparison of laboratory parameters between COVID‐19 patients with and without EBV reactivation

There was no significant difference in leukocytes count (*p* = .54), lymphocytes count (*p* = .96), platelets count (*p* = .97), serum levels of IL‐6 (*p* = .55), serum creatinine (*p* = .39), ferritin (*p* = .48), fibrinogen (*p* = .76), and triglyceride (*p* = .12) between patients with and without EBV reactivation (Table 1). Hemoglobin levels were significantly lower in patients with than without EBV reactivation (*p* = .007). Likewise, d‐dimer (*p* = .03) and total bilirubin levels (*p* = .006) were significantly higher in patients with EBV reactivation.

**TABLE 1:** Outcomes and laboratory tests in COVID‐19 patients with and without EBV reactivation

	Total (*n* = 217)	EBV reactivation negative group (*n* = 162)	EBV reactivation positive group (*n* = 55)	*p*
Death, *n* (%)	30 (14)	18 (11)	12 (22)	.08
Leukocyte, ×109/L, median (IQR)	5.29 (3.66, 7.82)	5.24 (3.65, 7.79)	5.33 (3.97, 7.83)	.54
Hb, g/L, median (IQR)	127 (117, 138)	129 (118.25, 140)	122 (111, 130.5)	.007
PLT, ×109/L, median (IQR)	180 (133, 258)	179 (135.25, 252.75)	182 (125, 267.5)	.97
Neu, ×109/L, median (IQR)	3.57 (2.37, 6.21)	3.55 (2.28, 6.04)	3.69 (2.51, 6.62)	.35
Lymphocyte, ×109/L, median (IQR)	1.01 (0.7, 1.37)	1.01 (0.7, 1.37)	1.03 (0.72, 1.36)	.96
Fib, g/L, median (IQR)	4.7 (3.7, 5.9)	4.7 (3.7, 5.8)	4.6 (3.9, 6)	.76
d‐Dim, mg/L, median (IQR)	0.61 (0.35, 1.24)	0.56 (0.33, 1.15)	0.9 (0.49, 1.6)	.03
Tbil, µmol/L, median (IQR)	11.3 (9.2, 13.9)	10.95 (8.9, 13.4)	12.5 (10.2, 16.15)	.006
ALT, U/L, median (IQR)	29 (19, 49)	30.5 (18.25, 48.75)	27 (19.5, 52)	.87
AST, U/L, median (IQR)	33 (26, 44)	32 (26, 43)	35 (26, 46.5)	.49
BUN, mmol/L, median (IQR)	4.6 (3.4, 5.7)	4.6 (3.4, 5.5)	4.6 (3.45, 6.7)	.28
sCr, umol/L, median (IQR)	71.3 (60.3, 82.2)	71.95 (61.02, 82.68)	70.4 (58.4, 78.55)	.39
TG, mmol/L, median (IQR)	1.23 (0.94, 1.67)	1.15 (0.91, 1.63)	1.33 (1.07, 1.69)	.12
Ferritin, ng/ml, median (IQR)	568.54 (312.25, 1033.02)	574.52 (312.25, 1016.09)	561.25 (320.25, 1107.46)	.48
IL‐6, pg/ml, median (IQR)	7.66 (5.87, 9.95)	7.48 (5.78, 9.46)	8.01 (6.23, 10.21)	.55

Abbreviations: ALT, alanine transaminase; AST, aspartate aminotransferase; BUN, blood urea nitrogen; COVID‐19, coronavirus disease 2019; d‐Dim, d‐dimer; EBV, Epstein–Barr virus; Fib, fibrinogen; Hb, hemoglobin; IL‐6, interleukin‐6; IQR, interquartile range; Neu, neutrophil; PLT, platelet; sCr, serum creatinine; Tbil, total bilirubin; TG, triglyceride.

### 3.3 Effects of ganciclovir treatment in patients with COVID‐19

A total of 88 patients have received ganciclovir treatment. Table 2 summarized the baseline characteristics of the ganciclovir‐treated group and matched controls. No differences between the ganciclovir treatment group and matched controls included SOFA and APACHEII scores (Table 2).

**TABLE 2:** Demographics and baseline characteristics of COVID‐19 patients with or without ganciclovir treatment

	Total (*n* = 176)	Control group (*n* = 88)	Treated group (*n* = 88)	*p*
Age, years, mean (SD)	48.96 (12.24)	49.25 (12.12)	48.67 (12.42)	.75
Gender, male, *n* (%)	114	57 (64.8)	57 (64.8)	>.99
SOFA score, median (IQR)	1 (0, 2)	1 (0, 2)	1 (0, 2)	.41
APACHEII score, median (IQR)	3 (2, 5)	3 (2, 5)	3 (2, 5)	.45
Severity, *n* (%)				.39
Mild and moderate	129 (73)	65 (74)	64 (73)	
Severe	33 (19)	14 (16)	19 (22)	
Critical	14 (8)	9 (10)	5 (6)	
Respiratory support, *n* (%)				.19
None	78 (45)	42 (48)	36 (41)	
Intranasal oxygen inhalation	90 (51)	44 (50)	46 (53)	
High‐flow nasal cannula	2 (1)	1 (1)	1 (1)	
Noninvasive ventilation	4 (2)	0 (0)	4 (5)	
Invasive ventilation	1 (1)	1 (1)	0 (0)	
Fever, *n* (%)	164 (93)	80 (91)	84 (95)	.37
Temperature, median (IQR)	38.8 (38, 39)	38.9 (38, 39.15)	38.5 (38, 39)	.26
Coinfection, *n* (%)				
Influenza A	7 (4)	1 (1)	6 (7)	.12
Influenza B	7 (4)	1 (1)	6 (7)	.12
Tuberculosis, *n* (%)	5 (3)	3 (3)	2 (2)	>.99
Symptoms and signs				
Nasal stuffiness, *n* (%)	3 (2)	2 (2)	1 (1)	>.99
Nasal discharge, *n* (%)	4 (2)	3 (3)	1 (1)	.62
Sneezing, *n* (%)	3 (2)	3 (3)	0 (0)	.25
Sore throat, *n* (%)	4 (2)	2 (2)	2 (2)	>.99
Cough, *n* (%)	145 (83)	73 (83)	72 (83)	>.99
Sputum production, *n* (%)	72 (41)	33 (38)	39 (45)	.41
Chest tightness, *n* (%)	66 (38)	41 (47)	25 (29)	.02
Chest pain, *n* (%)	6 (3)	3 (3)	3 (3)	>.99
Hemoptysis, *n* (%)	2 (1)	1 (1)	1 (1)	>.99
Headache, *n* (%)	18 (10)	11 (12)	7 (8)	.47
Myalgia, *n* (%)	17 (10)	9 (10)	8 (9)	>.99
Acratia, *n* (%)	58 (33)	33 (38)	25 (29)	.28
Digestive symptoms, *n* (%)	14 (8)	7 (8)	7 (8)	>.99
Discomfort of eye, *n* (%)	0 (0)	0 (0)	0 (0)	>.99
Cyanosis, *n* (%)	2 (1)	2 (2)	0 (0)	.50
Rhonchial, *n* (%)	5 (3)	5 (6)	0 (0)	.06
Moist rales, *n* (%)	18 (10)	15 (17)	3 (3)	.007
Personal history				
Smokers, *n* (%)	6 (4)	4 (5)	2 (3)	.69
Alcohol, *n* (%)	5 (4)	3 (4)	2 (3)	>.99
Medical staff, *n* (%)	1 (1)	1 (1)	0 (0)	>.99
Cluster cases, *n* (%)	30 (17)	8 (9)	22 (26)	.01
LOS, median (IQR)	12 (9, 16)	12 (8, 14.25)	14 (10, 17)	.006
ICU LOS, median (IQR)	0 (0, 5)	0 (0, 3)	0 (0, 6.25)	.36

Abbreviations: APACHE II, acute physiology and chronic health evaluation II; COVID‐19, coronavirus disease 2019; ICU, intensive care unit; IQR, interquartile range; LOS, length‐of‐stay; SD, standard deviation; SOFA, sequential organ failure assessment.

#### 3.3.1 Effects on mortality

**FIGURE 3:**
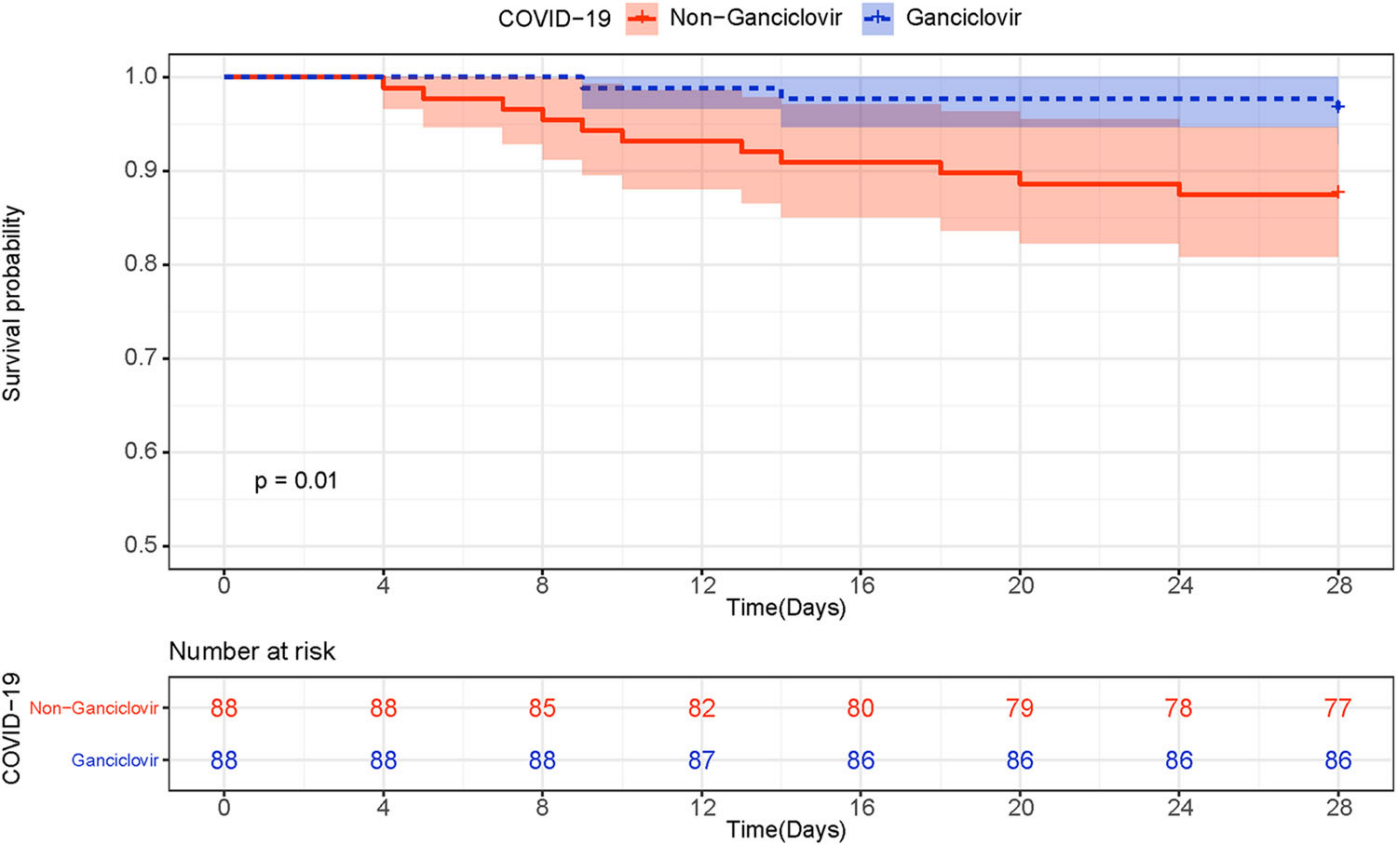
Kaplan–Meier survival curves for patients with ganciclovir therapy or who did not receive ganciclovir therapy. COVID‐19, coronavirus disease 2019

Ganciclovir treated patients had higher 28‐day survival rate than matched controls (0.98, 95% CI [0.95, 1.00] vs. 0.88, 95% CI [0.81, 0.95], *p* = .01) (Figure 3). In addition, there were no difference between groups in the use of antibiotics (88 [100%] vs. 84 [95%], *p* = .12) and glucocorticoids (19 [22%] vs. 28 [32%], *p* = .13).

#### 3.3.2 Effects on laboratory parameters

**FIGURE 4:**
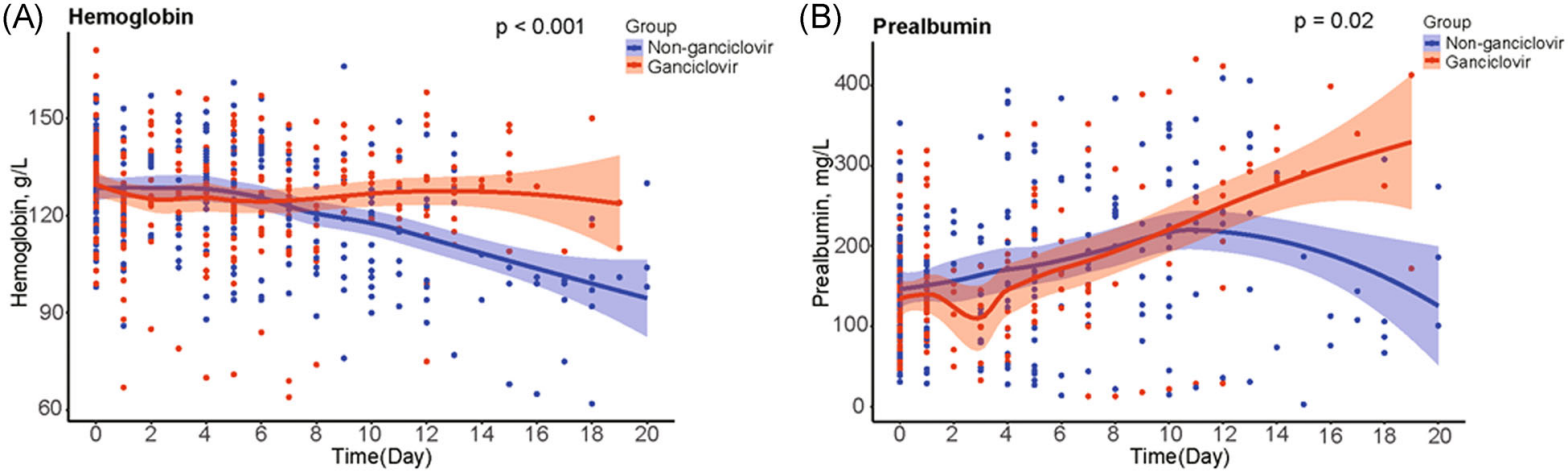
Comparison of hemoglobin and prealbumin between ganciclovir treated group and matched controls

**FIGURE 5:**
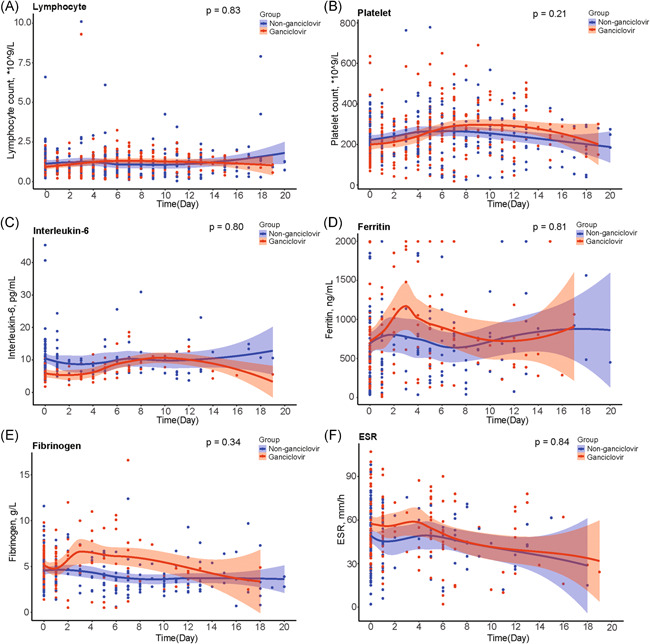
Comparison of lymphocyte count, platelet count, IL‐6, ferritin, fibrinogen, and ESR between ganciclovir treated group and matched controls. ESR, erythrocyte sedimentation rate; IL‐6, interleukin 6

Hemoglobin levels were significantly higher in ganciclovir treated patients than in controls (*p* < .001) (Figure 4A). Likewise, prealbumin levels were significantly higher in ganciclovir treated patients than in controls (*p* = .02) (Figure 4B). In contrast, there were no evidence for a similar difference between treated and control groups for lymphocyte count (*p* = .83), platelet count (*p* = .21), levels of IL‐6 (*p* = .80), ferritin levels (*p* = .81), fibrinogen levels (*p* = .34), and ESR (*p* = .84) (Figure 5).

### 3.4 Safety evaluation of ganciclovir therapy in COVID‐19 patients

**FIGURE 6:**
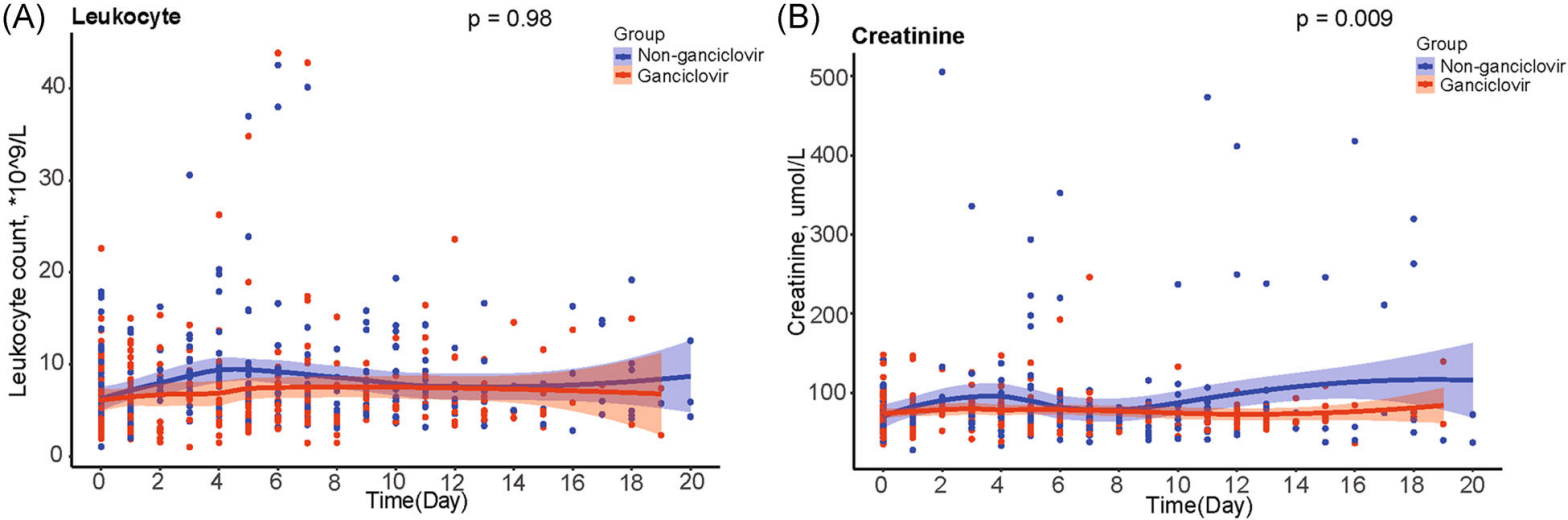
Comparison of leukocyte count and serum creatinine between ganciclovir treated group and matched controls

Compared to controls, ganciclovir‐treated patients had no significant changes in leukocyte count (*p* = .98) and had lower levels of serum creatinine (*p* = .009) (Figure 6).

## 4 DISCUSSION

This study demonstrated a high prevalence of EBV reactivation in patients with severe COVID‐19, which was even higher in nonsurvivors. Our study also suggested that treatment with ganciclovir may improve the survival of these patients.

EBV gene expression can be tested by immunohistochemistry, RT‐PCR, and nucleic acid sequence‐based amplification. EBV‐specific serological assays by enzyme‐linked immunosorbent assay or by the immunofluorescent assay are used for accurate confirmation of acute or convalescent EBV infection. EBV infection is accompanied by a series of regular serological reactions. VCA‐IgM first increased after the initial infection, while EBNA‐IgG appeared at least one month after the primary infection, at the same time as VCA‐IgG. VCA‐IgG can be used as a marker of the previous infection, a re‐elevation of VCA‐IgG can also be used as an indicator of EBV reactivation. EA‐IgG increased in the first infection and again in the pathological state of EBV reactivation. Serological tests for EBV‐specific antibodies are frequently used to identify EBV infection and to distinguish infection status. Normally, a profile of elevated EA‐IgG and VCA‐IgG was considered as the evidence for EBV reactivation.^11^ We defined EBV reactivation by the concomitant increase in VCA‐IgG and EA‐IgG.^11^

In this study, we found that the proportion of patients who were simultaneously positive for EA‐IgG and VCA‐IgG was significantly higher in severe COVID‐19 patients (25.3%) than in the healthy controls in North China (less than 4.22%).^20^ We also revealed that patients who died from COVID‐19 had a higher level of EA‐IgG than survivors. Moreover, ganciclovir treatment may improve the survival of severe COVID‐19 patients. Altogether, our study suggested a possibility that EBV may be reactivated and coparticipated in COVID‐19 mortality. The average sampling point was on the seventh day after the onset of the disease, suggesting that reactivation of EBV may be an early complication. In our study, the higher serum EA‐IgG concentrations in nonsurvivors suggested that EBV reactivation might be associated with poor prognosis. In another study, Le Balc'h et al.^21^ found that Herpesviridae reactivation was associated with old age, prolonged duration of mechanical ventilation, an increased intensive care unit length of stay, and a lower ratio of PaO_2_ to FiO_2_. However, given that the EBV‐reactivated group is enriched in older patients, the interaction between age and EBV‐reactivation on patient prognosis should be addressed in future studies. EBV is a double‐stranded DNA herpesvirus. EBV is mainly found in salivary glands and B lymphocytes during latent infection phase.^22^, ^23^ The virus load is mainly dependent on host immunity and immune suppression is strongly associated with EBV reactivation.^18^, ^23^ There is some evidence that COVID‐19 may be associated with dysregulated immune response and in particular lymphocytopenia, a feature of immune suppression which is commonly observed in patients with COVID‐19 and correlates with poor prognosis.^2^, ^8^, ^24^ Our results implied that the COVID‐19 pandemic might be a risk factor for the reactivation of herpes viruses. Reactivation may occur soon after or concurrently with SARS‐CoV‐2 virus infection. It might be necessary to pay attention to viral coinfection among COVID‐19 patients.

On the one hand, there is increasing evidence that in critically ill patients with COVID‐19, the mortality and multiple organ failure might be associated with an excessive upregulation of proinflammatory mediators.^4^, ^8^, ^24^, ^25^ On the other hand, EBV reactivation may further stimulate the inflammatory response and eventually trigger secondary hemophagocytic lymphohistiocytosis (sHLH).^9^, ^11^ sHLH is characterized by raging inflammatory cytokines and fatal multiple organ failure. The clinical symptoms of sHLH include persistent fever, hemocytopenia, and elevated ferritin, which are in consistent with the clinical phenotype of COVID‐19.^24^ Previous research showed a significantly higher incidence of anemia in nonsurvivors (26%) than in survivors (11%).^7^ In comparison with EA‐IgG‐negative patients, patients with evidence of EBV reactivation had significantly lower levels of hemoglobin and higher levels of total bilirubin. The EBV reactivation group was sicker than the nonreactivation group according to lower hemoglobin, higher d‐dimer and higher total bilirubin. Mortality was correspondingly higher in the EBV reactivation group (11% in EBV nonreactivation group vs. 22% in the EBV reactivation group). Therefore, EBV reactivation is likely a marker of severity of illness in COVD‐19 patients, which fits well with our understanding of reactivation of herpes viruses.^7^

This study was inconclusive with regard to potential CMV reactivation owing to the lack of serological specific antibodies. This issue should be addressed in future studies as CMV activation may occur simultaneously with EBV reactivation in critically ill patients, contributing to increased systemic and pulmonary inflammation.^10^ Ganciclovir could reduce lung injury by inhibiting CMV reactivation.^26^ Furthermore, we evaluated the effects of ganciclovir in patients with COVID‐19. There was no evidence for any difference in age, sex, the severity of illness, and respiratory support between ganciclovir‐treated patients and controls. In addition, other pharmacological therapies including antibiotic administration and glucocorticoid administration in the ganciclovir‐treated group and matched controls were similar. We found a −10% absolute reduction in 28‐day mortality in favor of ganciclovir. Ganciclovir also resulted in a significant improvement in hemoglobin levels, suggesting that blocking EBV reactivation may be associated with a reduction in EBV‐induced hemolysis and myelosuppression.^9^ Indeed, we found no evidence for a difference in the levels of inflammatory mediators such as IL‐6, platelet count, CRP, and ferritin between ganciclovir‐treated patients and controls. In another study, Limaye et al.^27^ also found that ganciclovir did not reduce IL‐6 levels among CMV‐seropositive adults with sepsis or trauma, which did not recommend using ganciclovir routinely. Ganciclovir is a commonly used antiviral medication. As a chemically synthesized guanine analogue, ganciclovir is clinically used against herpesvirus. Ganciclovir inhibits the binding of deoxyguanosine trivalent phosphate to DNA polymerase, resulting in the cessation of DNA prolongation, thereby preventing DNA virus replication.^28^ SARS‐CoV‐2 belongs to β‐coronavirus and is a positive‐stranded RNA virus.^29^ Although the underlying mechanism why ganciclovir treatment showed a survival benefit in COVID‐19 patients is largely unknown, it is unlikely that ganciclovir may have a direct inhibition of SARS‐CoV‐2. The survival benefits of ganciclovir treatment may be attributed to its effect on other viruses that were not tested in this study, such as the Roseoloviruses or the neurotropic alpha‐herpesviruses. Given the drug only targeting DNA virus, the possible therapeutic effects might be through the inhibition of EBV reactivation, which should be further explored in laboratories and clinical settings. Our study suggested that ganciclovir was safe in patients with COVID‐19, because there was no difference in leukocyte count between ganciclovir‐treated and ganciclovir‐free patients. In addition, ganciclovir treatment did not increase the serum creatinine levels in patients, indicating its safety for renal function.

This study has some limitations, mainly related to its retrospective design. First, the major limitation is that the diagnosis of EBV reactivation is based on serological antibodies as previously described.^11^ We could not analyze the diagnostic agreement between the serological method and PCR assay, because EBV nucleic acid testing was only performed in a very small proportion of patients during the early stage of the epidemic. Therefore, our findings only suggested a possibility of EBV reactivation but were not enough to draw a solid conclusion. Second, due to the retrospective nature of this study, sequential samples were not taken from patients before and after SARS‐Cov‐2 infection. Therefore, we are unable to trace the dynamic changes of serological antibodies during the course of COVID‐19. There is a risk of overestimating the prevalence of EBV reactivation by relying only on cross‐sectional data. Third, the survival benefits of ganciclovir treatment may be attributed to its effect on other viruses that were not tested in this study, such as the Roseoloviruses or the neurotropic alpha‐herpesviruses. Because this retrospective study had a small size population, the finding of EBV reactivation in COVID‐19 patients and ganciclovir treatment associated with improved survival rate was only exploratory and hypothesis‐generating. Thus, the mechanisms by which ganciclovir treatment cause improved survival remains to be further confirmed.

In conclusion, EBV reactivation may be frequent in COVID‐19 patients and may be associated with severity of illness and poor outcome. Whether treatment with ganciclovir may decrease the mortality of COVID‐19 patients complicated with EBV reactivation warrants to be addressed in a placebo‐controlled randomized trial.

## CONFLICT OF INTERESTS

The authors declare no conflict of interest.

## AUTHOR CONTRIBUTIONS

Mei Meng and Sheng Zhang were responsible for statistical analysis and figures construction. Mei Meng, Xuan Dong, Wenqing Sun, and Ranran Li wrote this manuscript and organized tables and figures. Yunfeng Deng and Wenzhe Li were responsible for medical information collection. Ranran Li, Zhixiong Wu, and Wenzhe Li were responsible for data extraction and verification. Djillali Annane and Dechang Chen designed and guided this study. All authors reviewed and approved the final version.

## Data Availability

The data that support the findings of this study are available from the corresponding author on reasonable request. After the publication of the study findings, the data will be available for others to request. Participant data without names and identifiers will be made available after approval from the corresponding author. The research team will provide an email address for communication once the data are approved to be shared with others.
